# Beyond Classical Diabetes Insipidus: A Retrospective Case Series Illustrating the Diagnostic Complexity and Diverse Etiologies of Polyuria-Polydipsia Syndrome

**DOI:** 10.7759/cureus.112989

**Published:** 2026-07-20

**Authors:** Debarchan Jena, Shamli Mishra, Sonali Palai, Bijay K Sahoo, Anwesha Mohanty, Bhabani S Dhal, Satyaprada Mishra, Sudhiranjan Pattanaik

**Affiliations:** 1 Endocrinology, Srirama Chandra Bhanja (S.C.B.) Medical College and Hospital, Cuttack, IND

**Keywords:** central diabetes insipidus, copeptin, nephrogenic diabetes insipidus, polyuria-polydipsia syndrome, primary polydipsia, water deprivation test

## Abstract

Background

Polyuria-polydipsia syndrome encompasses a heterogeneous group of disorders, including central diabetes insipidus (CDI), nephrogenic diabetes insipidus (NDI), and primary polydipsia. Despite presenting with similar clinical manifestations, these conditions differ markedly in their underlying pathophysiology, prognosis, and management. Distinguishing between them remains clinically challenging, particularly in patients with partial disease and in resource-limited settings where newer diagnostic biomarkers such as copeptin are not routinely available. This study highlights the diagnostic complexity and diverse etiologies of polyuria-polydipsia syndrome through a retrospective case series.

Methods

We conducted a retrospective observational study of patients presenting with polyuria-polydipsia syndrome at a tertiary care center over a five-year period. Clinical, biochemical, and radiological data were retrieved from medical records. Patients underwent diagnostic evaluation based on clinical indication, including assessment of serum and urine osmolality and a supervised water deprivation test with or without a desmopressin challenge, as appropriate. Additional imaging and genetic investigations were performed where clinically indicated. Patients were classified as having CDI, NDI, or primary polydipsia based on the available clinical, biochemical, radiological, and, where applicable, genetic findings in accordance with standard diagnostic criteria.

Results

A total of seven patients were included, demonstrating diverse etiologies: complete CDI (n = 1), CDI associated with pituitary stalk interruption syndrome (n = 1), partial CDI (n = 1), NDI with a possible CLCN5 gene variant (n = 1), primary polydipsia (n = 1), pheochromocytoma presenting as a diabetes insipidus mimic (n = 1), and CDI secondary to postoperative and post-radiotherapy craniopharyngioma with panhypopituitarism (n = 1). Despite similar presenting complaints, each case exhibited distinct biochemical and radiological profiles. The water deprivation test followed by a desmopressin challenge enabled definitive diagnosis. Etiology-specific management resulted in significant clinical improvement across the cohort.

Conclusion

Polyuria-polydipsia syndrome represents a diagnostically challenging entity with diverse underlying etiologies. A structured approach integrating clinical evaluation, biochemical testing, dynamic assessment, and imaging remains effective for accurate diagnosis, even in the absence of advanced biomarkers. Recognition of atypical and reversible causes is essential to avoid misdiagnosis and ensure appropriate, targeted management.

## Introduction

Polyuria-polydipsia syndrome represents a spectrum of disorders characterized by excessive urine output and increased fluid intake, most commonly including central diabetes insipidus (CDI), nephrogenic diabetes insipidus (NDI), and primary polydipsia. Although these entities share similar clinical presentations, they differ fundamentally in their underlying pathophysiology and management strategies, making accurate differentiation essential for appropriate treatment [1-3].

The distinction between these conditions, particularly partial CDI and primary polydipsia, remains clinically challenging due to overlapping biochemical and clinical features. Misclassification can lead to inappropriate therapy, including the risk of hyponatremia with desmopressin use in primary polydipsia [4,5]. While newer diagnostic modalities such as copeptin-based assays have improved diagnostic accuracy, their limited availability in resource-constrained settings restricts their routine use.

In such settings, a structured diagnostic approach incorporating clinical assessment, biochemical evaluation, dynamic testing, and imaging remains the cornerstone of diagnosis. Conventional methods, including the water deprivation test and desmopressin challenge, continue to play a pivotal role in differentiating various etiologies of hypotonic polyuria.

Despite existing diagnostic frameworks, the polyuria-polydipsia spectrum remains heterogeneous, with occasional atypical and reversible causes that further complicate evaluation. There is a paucity of real-world data highlighting this diversity, particularly from resource-limited settings.

In this study, we present a retrospective case series of seven patients with polyuria-polydipsia syndrome who exhibited comparable clinical presentations but were found to have diverse underlying etiologies following systematic evaluation. This study aims to illustrate the diagnostic challenges and diverse etiologies of polyuria-polydipsia syndrome, highlight the utility of conventional diagnostic approaches in resource-limited settings, and emphasize the importance of individualized evaluation for accurate diagnosis and appropriate management.

## Materials and methods

This retrospective observational study was conducted in the Department of Endocrinology at a tertiary care center. Ethical approval for the study was obtained from the Institutional Ethics Committee (IEC No. 2529/22.04.26). Patients presenting with polyuria-polydipsia syndrome who underwent comprehensive diagnostic evaluation over the past five years were included. Data were retrieved from hospital medical records, including clinical details, laboratory parameters, imaging findings, and treatment outcomes. A total of seven patients fulfilling the eligibility criteria were identified and included in the final analysis. The detailed inclusion and exclusion criteria are summarized in Table 1.

**Table 1:** Inclusion and exclusion criteria of the study population.

Inclusion criteria	Exclusion criteria
Patients presenting with clinical features suggestive of polyuria-polydipsia syndrome	Patients with incomplete or unavailable medical records
Documented polyuria, defined as urine output > 50 mL/kg over 24 hours	Polyuria secondary to uncontrolled diabetes mellitus
Availability of complete clinical evaluation records	Patients with chronic kidney disease causing impaired urinary concentrating ability
Availability of biochemical evaluation including serum sodium, serum osmolality, and urine osmolality	Polyuria attributable to osmotic diuresis from non-endocrine causes
Patients who underwent appropriate diagnostic evaluation including water deprivation test and/or desmopressin challenge where indicated	Drug-induced polyuria or diuresis
Availability of imaging studies and/or genetic evaluation wherever clinically indicated	Patients in whom a definitive etiological classification of polyuria-polydipsia syndrome could not be established

The evaluation of patients followed a structured and stepwise diagnostic approach to ensure accurate etiological classification [1]. The initial step involved confirmation of true polyuria, defined as a urine output exceeding 50 mL/kg over a 24-hour period. Patients with urine output below this threshold were excluded from further endocrine evaluation and were instead considered for alternative urological causes.

Once polyuria was confirmed, urine osmolality was assessed to identify hypotonic polyuria. A urine osmolality value of less than 800 mOsm/kg was considered indicative of hypotonic polyuria and prompted further evaluation. Subsequently, serum sodium and plasma osmolality were measured to provide preliminary diagnostic direction. Hyponatremia (serum sodium < 135 mmol/L) was suggestive of primary polydipsia, whereas hypernatremia (serum sodium > 147 mmol/L) favored a diagnosis of diabetes insipidus (DI), either central or nephrogenic. Patients with serum sodium levels in the intermediate range (136-146 mmol/L) required further dynamic testing.

A supervised water deprivation test was then performed to evaluate urinary concentrating ability. All patients who underwent water deprivation testing were evaluated using a standardized institutional protocol. The test was conducted under continuous medical supervision, with body weight, urine output, urine osmolality, serum sodium, and plasma osmolality assessment at baseline and every two hours thereafter. The test was terminated upon reaching predefined safety endpoints, including ≥3% loss of body weight, serum sodium > 145 mmol/L, or a plateau in urine osmolality on two consecutive measurements, defined as an increase of <30 mOsm/kg (or <10%) between two consecutive measurements despite ongoing dehydration. Patients who achieved a urine osmolality greater than 800 mOsm/kg during dehydration were considered to have an intact vasopressin axis, effectively excluding DI and supporting a diagnosis of primary polydipsia.

In patients who failed to adequately concentrate urine (urine osmolality < 800 mOsm/kg), further differentiation was performed based on urine osmolality thresholds. Values between 300 and 800 mOsm/kg were considered indeterminate and suggestive of either partial CDI or chronic primary polydipsia, while values less than 300 mOsm/kg indicated significant impairment in urinary concentrating ability, as seen in complete DI.

All such patients subsequently underwent a desmopressin challenge test. A standardized dose of 5 U of desmopressin was administered subcutaneously (SC), except in the two-year-old child, who received vasopressin at a dose of 1 U/m² (0.5 U SC), and urine osmolality was reassessed approximately 1-2 hours after administration to determine renal responsiveness. Among patients with intermediate urinary concentrating ability (urine osmolality 300-800 mOsm/kg following water deprivation), the percentage change in urine osmolality after desmopressin administration was used to differentiate partial CDI from primary polydipsia. An increase in urine osmolality of >9% following desmopressin was considered suggestive of partial CDI, whereas an increase of <9% favored primary polydipsia. In patients with severe impairment of urinary concentrating ability (urine osmolality < 300 mOsm/kg), an increase in urine osmolality of more than 50% after desmopressin administration confirmed complete CDI, while an increase of less than 50% was consistent with NDI.

Copeptin-based evaluation represents a newer diagnostic approach for differentiating causes of hypotonic polyuria. Measurement of baseline plasma copeptin helps identify NDI, with a baseline copeptin concentration > 21.4 pmol/L considered diagnostic due to preserved or elevated vasopressin secretion in the presence of renal resistance. In patients with baseline copeptin levels < 21.4 pmol/L, a hypertonic saline-stimulated copeptin test is recommended. Following elevation of plasma sodium to >150 mmol/L under close monitoring, a stimulated copeptin concentration ≥ 4.9 pmol/L supports the diagnosis of primary polydipsia, whereas values < 4.9 pmol/L indicate complete or partial CDI due to impaired vasopressin secretion.

However, copeptin measurement was not available at our center during the study period; therefore, final etiological classification in our cohort was based on clinical assessment, biochemical evaluation, supervised water deprivation testing, desmopressin response, and additional investigations including magnetic resonance imaging (MRI) of the hypothalamic-pituitary region, abdominal imaging, and genetic testing wherever clinically indicated to identify structural, secondary, or hereditary causes.

Statistical analysis was performed using descriptive methods given the small sample size. Continuous variables were expressed as ranges, and categorical variables as frequencies and percentages. Case-wise descriptive analysis was also undertaken.

## Results

Case 1

A 40-year-old male patient presented with an insidious onset of profound polyuria and polydipsia for approximately one year, associated with significant nocturia that disrupted his daily activities. There were no symptoms suggestive of urinary tract infection, diabetes mellitus, significant weight loss, renal failure, thyroid illness, psychiatric illness, or exogenous drug exposure. He denied headache, visual blurring, postural dizziness, head trauma, or any prior history of intracranial tumor or cranial irradiation. Family history was non-contributory. On examination, his blood pressure was 110/70 mm Hg, and general as well as systemic examinations were unremarkable.

Following admission, polyuria was documented (80-90 mL/kg/day), and routine investigations including complete blood count, liver and renal function tests, thyroid profile, 8 AM cortisol, and calcium profile were within normal limits. Further evaluation revealed low urine osmolality (88 mOsm/kg) (reference range: 300-900 mOsm/kg) with high-normal serum osmolality (295 mOsm/kg) (reference range: 275-295 mOsm/kg). In view of suspected DI, a formal water deprivation test with desmopressin challenge was performed under close supervision. The patient was deprived of fluids with serial monitoring of body weight, urine output, urine osmolality, and periodic assessment of serum osmolality and sodium. The test was terminated upon reaching an adequate dehydration endpoint (serum Na^+^ > 145 mmol/L) following which desmopressin (5 U) was administered SC, and urine osmolality was reassessed. The test demonstrated a minimal rise in urine osmolality during dehydration, followed by a robust increase (>70%) after desmopressin administration, confirming CDI.

**Figure 1:**
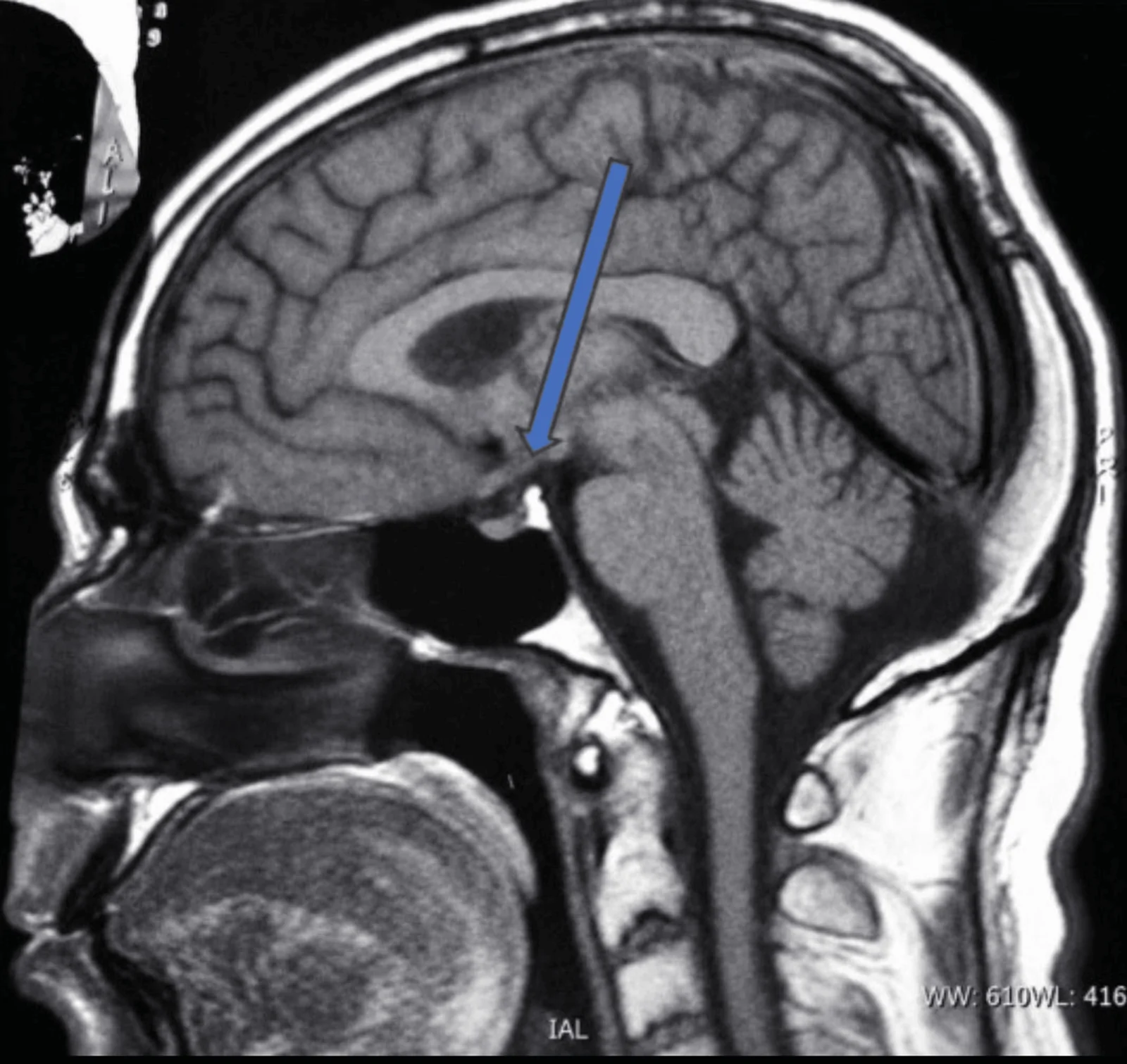
Sagittal T1-weighted MRI of the sella showing absence of the normal posterior pituitary bright spot (arrow), consistent with central diabetes insipidus. MRI: magnetic resonance imaging

MRI of the sella revealed the absence of the characteristic posterior pituitary bright spot on T1-weighted sequences (Figure 1).

Oral desmopressin (200 mcg/day) was started, which yielded rapid and sustained symptomatic resolution with restoration of euvolemia. On follow-up, urine output decreased to 30-40 mL/kg/day.

Case 2

A 35-year-old male patient presented with complaints of delayed onset of secondary sexual characteristics and infantile genitalia. He also reported poor height gain since childhood compared to his peers. There was no history of headache, visual disturbances, seizures, head trauma, or prior cranial irradiation. He denied symptoms suggestive of other hormonal deficiencies.

On examination, his height was 143 cm (<3rd centile), and his weight was 60 kg. Sexual maturity rating (SMR) was A2P3. Stretched penile length was 7.8 cm (<−2.5 SD for age), and bilateral testicular volume was 10 mL. General and systemic examinations were unremarkable. Routine laboratory investigations were within normal limits. Hormonal evaluation revealed hypogonadotropic hypogonadism, with luteinizing hormone (LH) 0.2 mIU/mL (reference range: 1.7-8.6 mIU/mL), follicle-stimulating hormone (FSH) 1.28 mIU/mL (reference range: 1.5-12.4 mIU/mL), and a suboptimal response to triptorelin stimulation (peak LH 3.3 mIU/mL; expected pubertal response > 5-8 mIU/mL). Serum testosterone was low (8 AM testosterone: 52 ng/dL; reference range: 300-1,000 ng/dL). Prolactin (8.98 ng/mL; reference range: 4-15 ng/mL) and 8 AM cortisol (10.5 µg/dL; reference range: 5-25 µg/dL) were within normal limits, while insulin-like growth factor-1 (IGF-1) was reduced (25 ng/mL; reference range for age approximately 116-358 ng/mL; <5th centile for age), suggestive of multiple anterior pituitary hormone deficiencies.

During evaluation, polyuria was documented (50-60 mL/kg/day). Biochemical assessment showed serum osmolality of 295 mOsm/kg (reference range: 275-295 mOsm/kg) and urine osmolality of 150 mOsm/kg (reference range: 300-900 mOsm/kg), raising suspicion for DI. Given the clinical and biochemical profile, a presumptive diagnosis of CDI was made.

**Figure 2:**
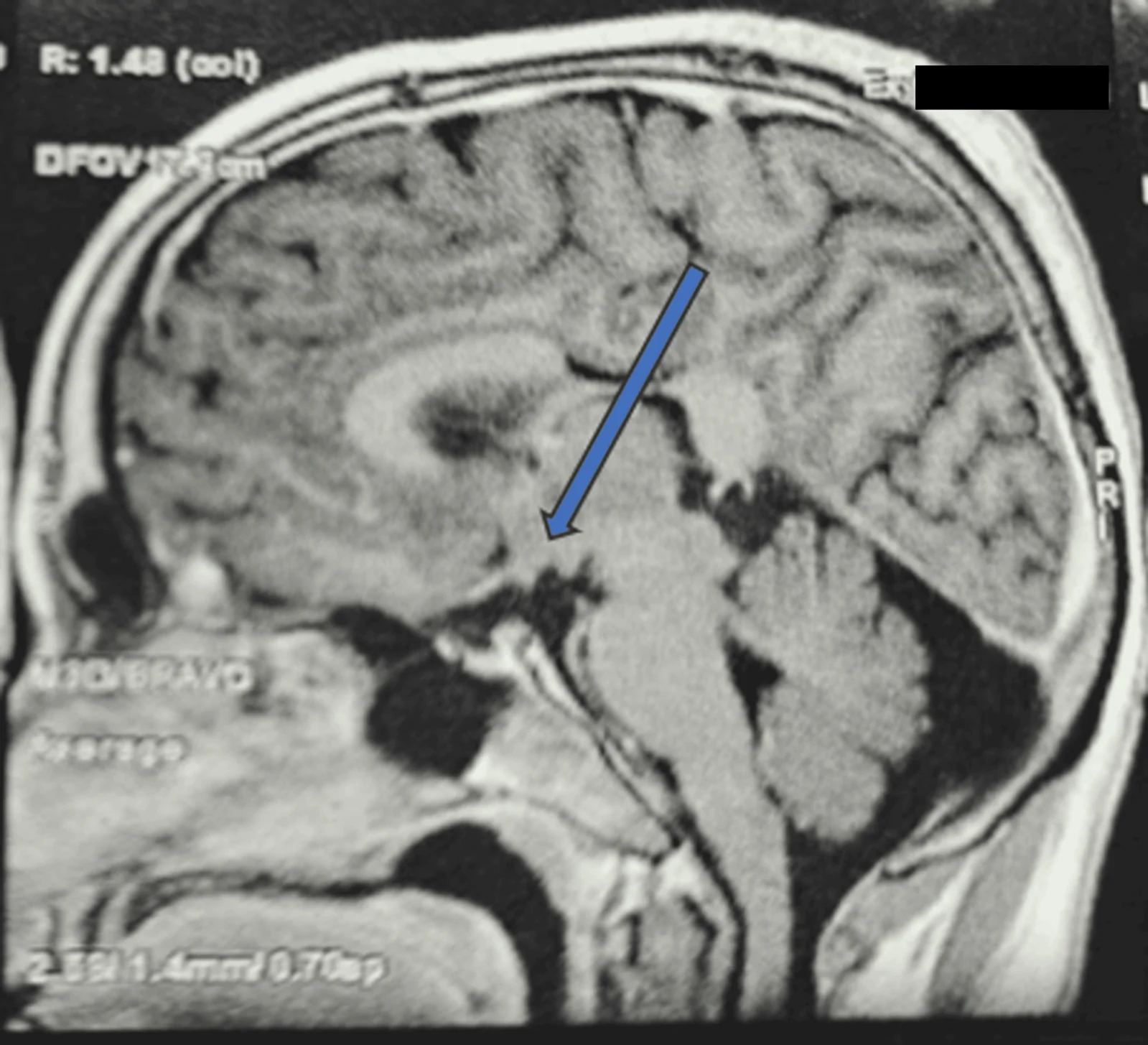
Sagittal T1-weighted MRI of the hypothalamic-pituitary region demonstrating absence of the normal posterior pituitary bright spot, with an ectopic anterior pituitary located along the median eminence and non-visualization of the pituitary stalk, findings consistent with pituitary stalk interruption syndrome (PSIS). MRI: magnetic resonance imaging

MRI of the hypothalamic-pituitary region revealed non-visualization of the posterior pituitary bright spot, with an ectopic anterior pituitary and interrupted pituitary stalk, consistent with pituitary stalk interruption syndrome (PSIS) (Figure 2).

A formal water deprivation test was not performed because the structural diagnosis and associated multiple anterior pituitary hormone deficiencies made the diagnosis of CDI clinically evident, and additional dynamic testing was considered unnecessary. Genetic evaluation by whole-exome sequencing did not reveal any pathogenic mutation. The patient was initiated on appropriate hormonal replacement therapy, including androgen replacement and other indicated deficiencies, along with oral desmopressin, which resulted in significant improvement in polyuria.

Case 3

A 38-year-old female patient, recently diagnosed with type 2 diabetes mellitus four months prior, was admitted for evaluation of persistent polyuria despite adequate glycemic control. She reported significant polyuria with associated nocturia for the past eight months, along with nocturnal thirst and frequent water intake. General and systemic examinations were unremarkable. During hospitalization, polyuria was documented (70-80 mL/kg/day).

**Figure 3:**
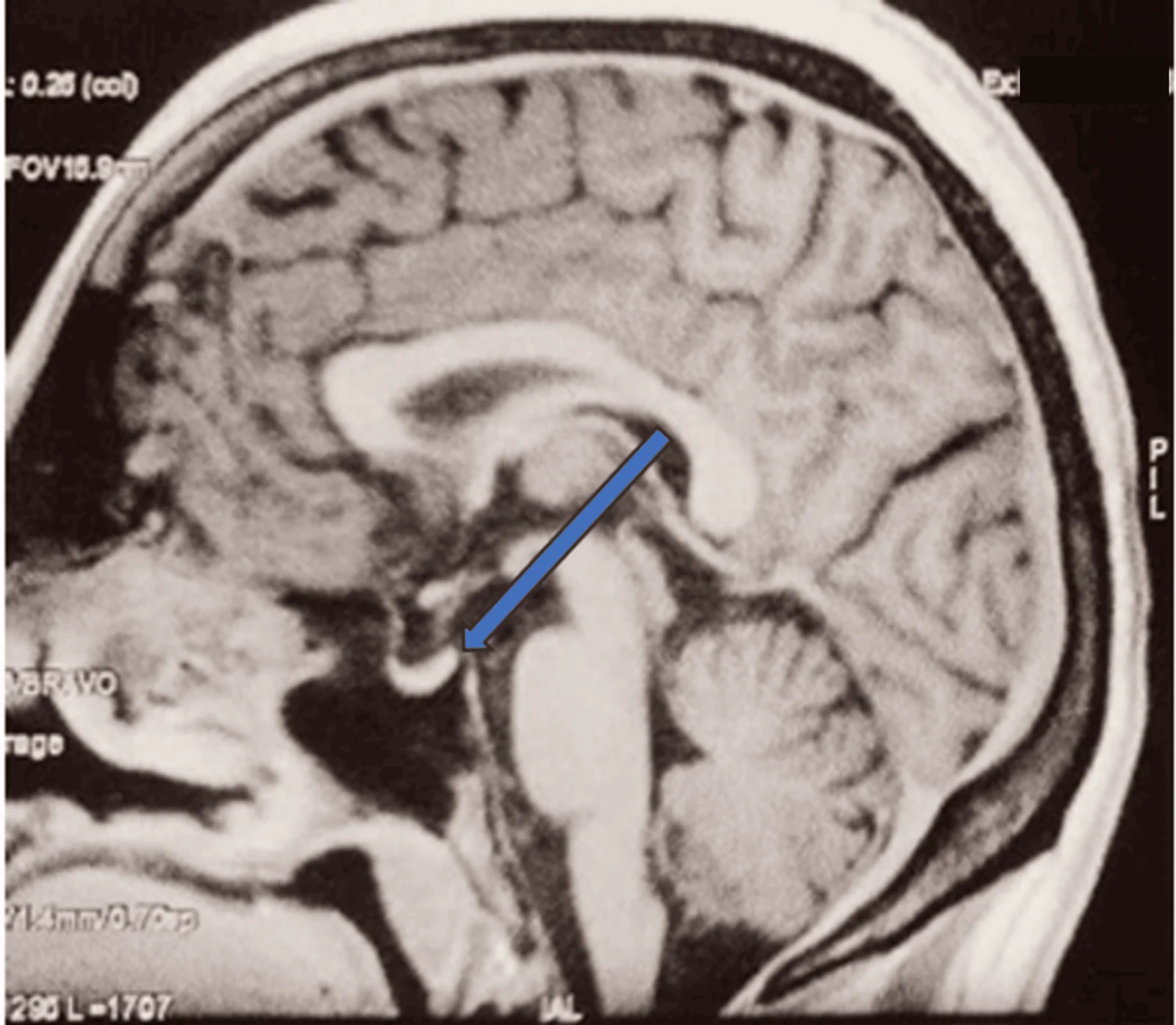
Sagittal T1-weighted MRI of the hypothalamic-pituitary region demonstrating an empty sella turcica, characterized by a flattened pituitary gland along the sellar floor with cerebrospinal fluid filling the sella, consistent with a structural etiology of partial central diabetes insipidus. MRI: magnetic resonance imaging

Baseline investigations revealed low urine osmolality (128 mOsm/kg) with serum osmolality of 284 mOsm/kg, raising suspicion for a water balance disorder. A formal water deprivation test followed by a desmopressin challenge was performed under close supervision. During the dehydration phase, there was an intermediate rise in urine osmolality, which increased significantly following desmopressin administration, suggestive of partial CDI. MRI of the hypothalamic-pituitary region demonstrated an empty sella turcica, supporting a structural basis for the disorder (Figure 3).

The patient was initiated on oral desmopressin (200 mcg/day), resulting in marked symptomatic improvement with a reduction in urine output and nocturia. She remains on regular follow-up with a sustained clinical response.

Case 4

**Figure 4:**
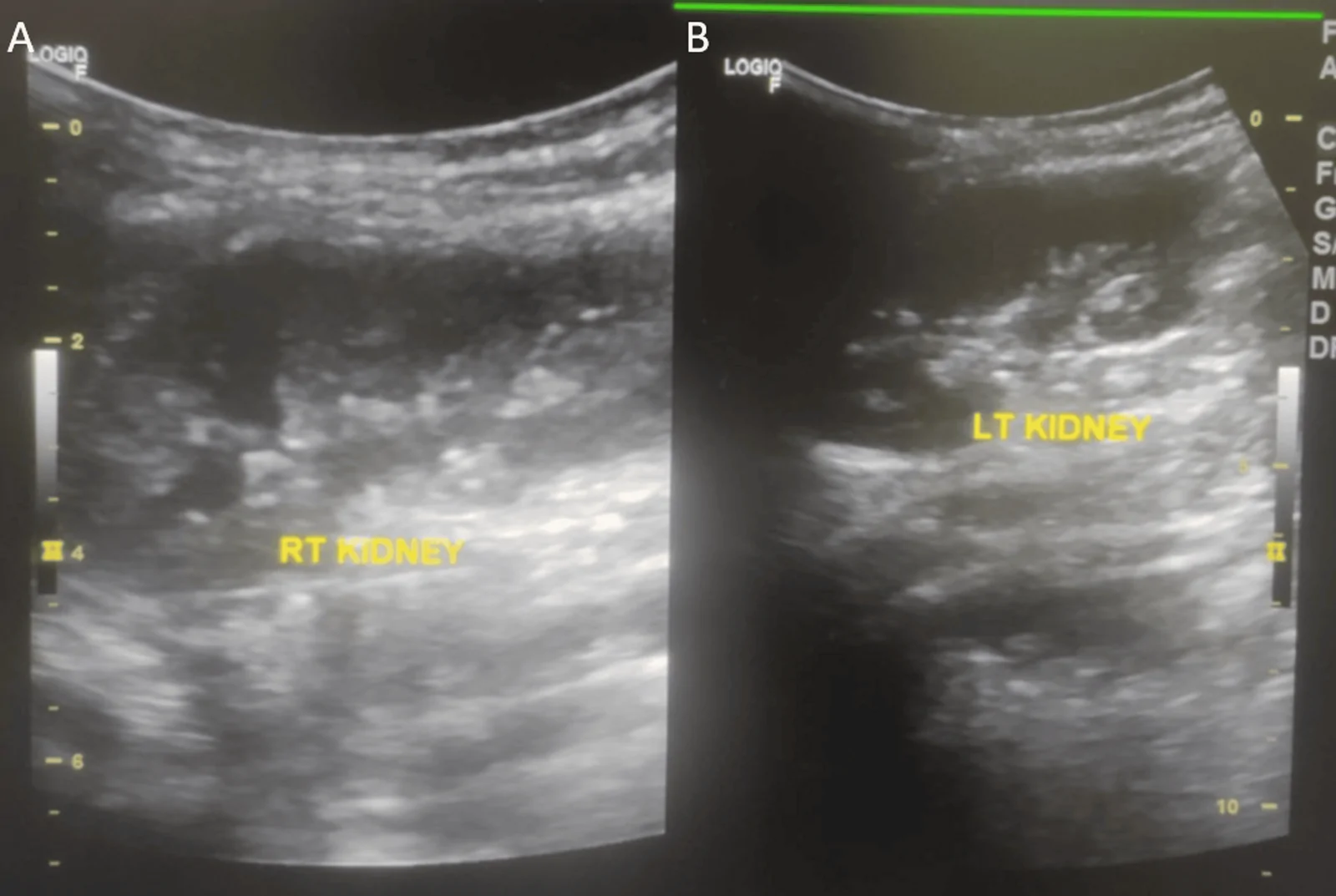
(A, B) Ultrasonography of the abdomen and pelvis demonstrating normal sonographic appearance of both kidneys with preserved renal morphology and corticomedullary differentiation. No evidence of nephrocalcinosis, hydronephrosis, renal calculi, or structural renal abnormality was identified, thereby excluding secondary renal structural causes of polyuria.

A two-year-old child was referred for evaluation of severe, unrelenting polyuria and polydipsia for the last six months. The mother gave a history of the child being diagnosed with primary pulmonary tuberculosis at the age of six months, when he was being evaluated at the Department of Pediatrics for pyrexia of unknown origin. He was started empirically on antitubercular therapy (ATT) for a period of six months; however, his current symptoms developed after 5-6 months of stoppage of these drugs. There was no history suggestive of central nervous system (CNS) infection, head trauma, tumor, or radiation to the head and neck, and no history of graveluria, abdominal pain, constipation, frothy urine, or recurrent muscle weakness. There was no similar family history. On examination, the child was hemodynamically stable with normal systemic findings. He did not exhibit any clinical features of rickets or nephrocalcinosis. Anthropometric measurements revealed a height of 83 cm (third centile) and a weight of 11 kg (10th centile). Bone age corresponded to chronological age (two years). Routine laboratory investigations, including arterial blood gas analysis and urine pH, were within normal limits. Ultrasonography of the abdomen and pelvis showed no abnormalities (Figure 4).

Baseline investigations revealed urine osmolality of 69 mOsm/kg (reference range: 300-900 mOsm/kg) and serum osmolality of 295 mOsm/kg (reference range: 275-295 mOsm/kg). A water deprivation test demonstrated persistently low urine osmolality with less than 30% increase following vasopressin administration (1 U/m^2^ = 0.5 U SC), consistent with NDI.

Whole-exome sequencing identified a mutation in exon 4 of the CLCN5 gene, classified as a variant of uncertain significance (VUS), suggestive of Dent disease. The patient was started on Tab. hydrochlorothiazide (3 mg/kg/day) along with Tab. amiloride (5 mg), and there was only partial improvement in his symptoms, which goes in favor of NDI.

Case 5

A 20-year-old male fitness enthusiast presented with complaints of habitual excessive fluid intake and excessive urination for the last one year, along with weight loss of around 8 kg over the last eight months. He described his thirst as so intense that he experienced severe discomfort whenever he tried to restrict his water intake. On inquiry, he gave a history of intermittent usage of Tab. amitriptyline and Tab. valproate for his migraine episodes for the past one year. There was no history of hepatic or renal failure, sickle cell disease, headache, visual disturbances, other CNS symptoms, burning micturition, or abdominal pain. On examination, his blood pressure was 100/60 mm Hg, and general and systemic examination were normal. Routine evaluation did not reveal any abnormality. Baseline urine osmolality was 315 mOsm/L and serum osmolality 294 mOsm/L. Water deprivation elicited a brisk and substantial rise in urine osmolality, confirming an intact vasopressin axis. Subsequent desmopressin administration produced only minimal further response, supporting primary polydipsia. Based on the above findings, a diagnosis of primary polydipsia was made, and the patient was managed conservatively, with a focus on behavioral fluid restriction.

Case 6

A 20-year-old female patient was admitted for evaluation of polyuria and polydipsia for the last one year. She drank nearly 5-6 L of water per day and had urine output in the range of 6-7 L/day, which resulted in multiple nocturnal awakenings and disturbed daily activities. She consulted multiple physicians for these complaints, but no cause was found, and she was advised to undergo behavioral modification. On examination, her heart rate was 110 beats per minute, and her blood pressure was persistently elevated at 170/90 mm Hg on repeated measurements. Initial laboratory investigations were unremarkable. Further evaluation revealed a urine osmolality of 110 mOsm/kg and a serum osmolality of 295 mOsm/kg.

Fundoscopic examination revealed grade IV hypertensive retinopathy. Two-dimensional echocardiography showed no abnormalities. Ultrasonography of the abdomen and pelvis identified a heterogeneously enhancing suprarenal lesion over the right kidney. Subsequent contrast-enhanced computed tomography (CECT) of the abdomen with adrenal protocol demonstrated a 6.4 × 6.5 × 4.5 cm intensely enhancing heterogeneous right adrenal mass with central necrosis, with absolute washout of 50% and relative washout of 30%, suggestive of pheochromocytoma. Biochemical evaluation revealed markedly elevated urinary catecholamine metabolites, with metanephrine levels of 99.88 µg/day (normal < 276.1 µg/day) and normetanephrine levels of 6,710 µg/day (normal < 549.6 µg/day). In view of the severe uncontrolled hypertension and strong clinical suspicion of pheochromocytoma, a formal water deprivation test was deferred because of potential safety concerns.

Following appropriate preoperative preparation, the patient underwent surgical resection of the adrenal mass. Antihypertensive medications were discontinued immediately postoperatively. Her polyuria and polydipsia resolved completely within 3-4 days after surgery without desmopressin therapy, effectively excluding DI and supporting pheochromocytoma as a reversible DI mimic.

Case 7

An 11-year-5-month-old female child, a known case of postoperative and post-radiotherapy craniopharyngioma, was admitted for comprehensive hormonal evaluation. She had undergone surgical resection of the tumor two years prior and, due to residual disease, subsequently received 30 cycles of conventional radiotherapy (total dose: 54 Gy).

During the current admission, her mother reported a six-month history of progressive polyuria and polydipsia, associated with multiple nocturnal awakenings and increased water intake. There was no history suggestive of infection, uncontrolled diabetes mellitus, or drug-induced diuresis.

On general examination, the patient had mild pallor. Fundoscopic evaluation revealed bilateral optic atrophy, consistent with prior optic pathway involvement. No other significant abnormalities were noted on systemic examination. Anthropometric assessment showed short stature and underweight status, with height of 136 cm (3rd-10th percentile) and weight of 28 kg (3rd-10th percentile). Mid-parental height was 151.75 cm, with a target height range of 148-158 cm and a projected adult height of 148.5 cm. SMR was prepubertal (A1P1B1). Bone age was delayed by eight years.

Baseline laboratory investigations were unremarkable. However, detailed hormonal evaluation revealed multiple anterior pituitary hormone deficiencies, including secondary hypothyroidism (FT4: 0.16 ng/mL; reference range: 0.8-1.8 ng/dL), hypocortisolism (8 AM cortisol: <0.5 µg/dL; reference range: 5-25 µg/dL), growth hormone deficiency suggested by low IGF-1 levels (25.5 ng/mL; reference range for age approximately 120-550 ng/mL; <5th percentile for age), and hypogonadotropic hypogonadism (LH: 0.001 mIU/mL; reference range: 0.3-6.2 mIU/mL, FSH: 0.40 mIU/mL; reference range: 1-8 mIU/mL), consistent with evolving panhypopituitarism.

Polyuria was documented at 130-140 mL/kg/day. Biochemical evaluation showed a low urine osmolality (142 mOsm/kg) in the presence of normal-high serum osmolality (295 mOsm/kg), suggestive of a defect in urinary concentration. Given the characteristic postoperative and post-radiotherapy setting, together with the typical biochemical profile and associated panhypopituitarism, a formal water deprivation test was not performed because the diagnosis of CDI was considered clinically apparent.

The patient was initiated on oral desmopressin along with appropriate hormone replacement therapy, including glucocorticoid and thyroid hormone supplementation. This resulted in significant clinical improvement, with marked reduction in polyuria and polydipsia and improved overall well-being.

The clinical, biochemical, imaging, and genetic profiles of all seven patients have been summarized, highlighting the heterogeneity and diagnostic complexity within the polyuria-polydipsia spectrum (Table 2).

**Table 2:** Summary of clinical presentation, diagnostic evaluation, etiology, management, and outcomes of patients with polyuria-polydipsia syndrome. AM cortisol: morning cortisol; CDI: central diabetes insipidus; CLCN5: chloride voltage-gated chloride channel 5 gene; DI: diabetes insipidus; F: female; FSH: follicle-stimulating hormone; IGF-1: insulin-like growth factor-1; LH: luteinizing hormone; M: male; NDI: nephrogenic diabetes insipidus; PSIS: pituitary stalk interruption syndrome; T2DM: type 2 diabetes mellitus; VUS: variant of uncertain significance

Case	Age (in years)/sex	Presentation	Urine osmolality (in mOsm/kg) (reference value: 300-900 mOsm/kg)	Serum osmolality (in mOsm/kg) (reference value: 275-295 mOsm/kg)	Water deprivation test	Desmopressin response	Additional laboratory findings (reference values)	Imaging findings	Genetic findings	Final diagnosis	Treatment	Outcome
1	40/M	Polyuria, polydipsia, nocturia (1 yr)	88	295	Minimal rise	>70% increase	Routine biochemical and hormonal evaluation within normal limits	Absent posterior pituitary bright spot	Not done	CDI	Oral desmopressin	Marked improvement
2	35/M	Delayed puberty, short stature + polyuria	150	295	Not performed	Presumed positive	LH: 0.2 mIU/mL (1.7-8.6 mIU/mL); FSH: 1.28 mIU/mL (1.5-12.4 mIU/mL); testosterone: 52 ng/dL (300-1,000 ng/dL); IGF-1: 25 ng/mL (116-358 ng/mL)	Ectopic pituitary, interrupted stalk (PSIS)	No pathogenic mutation	CDI (PSIS)	Hormone replacement + desmopressin	Improved
3	38/F	Polyuria despite controlled T2DM	128	284	Intermediate rise after dehydration	Significant increase (80% increase)	Routine biochemical evaluation within normal limits	Empty sella	Not done	Partial CDI	Oral desmopressin	Improved
4	2/M	Severe polyuria, polydipsia (6 months)	69	295	Minimal rise after dehydration	<30% increase	Arterial blood gas and urine pH within normal limits	Normal	CLCN5 exon 4 variant (VUS)	NDI (Dent disease spectrum)	Thiazide + amiloride	Partial response
5	20/M	Excess fluid intake, weight loss	315	294	Brisk rise	Minimal response (2% increase)	Routine biochemical evaluation within normal limits	Normal	Not done	Primary polydipsia	Behavioral therapy	Improved
6	20/F	Polyuria + hypertension	110	295	Not performed	Not applicable	Urinary metanephrine: 99.88 µg/day (<276.1 µg/day); normetanephrine: 6,710 µg/day (<549.6 µg/day)	Right adrenal mass (pheochromocytoma)	Not done	Pheochromocytoma (DI mimic)	Surgical resection	Complete resolution
7	11/F	Postoperative and post-radiotherapy craniopharyngioma with polyuria-polydipsia (6 months)	142	295	Not performed	Presumed positive	fT4: 0.16 ng/dL (0.8-1.8 ng/dL); 8 AM cortisol: <0.5 µg/dL (5-25 µg/dL); IGF-1: 25.5 ng/mL (120-550 ng/mL); LH: 0.001 mIU/mL (0.3-6.2 mIU/mL); FSH: 0.40 mIU/mL (1-8 mIU/mL)	Post-surgical changes in sellar region (craniopharyngioma)	Not done	CDI with panhypopituitarism	Desmopressin + hormone replacement	Marked improvement

## Discussion

In this case series, we evaluated seven patients presenting with polyuria-polydipsia syndrome and demonstrated that, despite strikingly similar clinical presentations, the underlying etiologies were markedly heterogeneous. This observation is consistent with prior literature, where CDI, NDI, and primary polydipsia frequently overlap clinically, often leading to diagnostic delays or misclassification [1,4,6]. However, our series further highlights this heterogeneity within a relatively small cohort, reinforcing the need for a structured diagnostic approach.

In Case 1, we observed classical complete CDI with a robust response to desmopressin and absence of the posterior pituitary bright spot. Similar findings have been widely reported, with Maghnie et al. demonstrating that loss of the neurohypophyseal bright spot is a sensitive marker of CDI [2,7]. Our findings are concordant with these observations and reaffirm that conventional MRI continues to play a pivotal adjunctive role alongside biochemical testing.

In Case 2, CDI was identified in association with PSIS, along with multiple anterior pituitary hormone deficiencies. PSIS is a rare congenital cause of hypopituitarism having variable posterior pituitary involvement. PSIS usually has a good clinical prognosis; however, untreated patients may develop severe features of underlying hormone deficiencies, like myxedema coma or adrenal insufficiency [3,8]. In contrast to some reports where CDI may be absent or delayed, our patient exhibited overt CDI, suggesting that posterior pituitary dysfunction in PSIS may be more clinically significant than traditionally perceived.

Case 3, representing partial CDI, illustrates the well-recognized diagnostic ambiguity between partial CDI and primary polydipsia. Prior studies have emphasized that partial CDI often falls into a “diagnostic gray zone,” with overlapping biochemical profiles [4,5,9]. Our findings align with these reports; however, the presence of a structural abnormality (empty sella) in our patient provides additional support for an organic etiology, a feature not consistently highlighted in earlier series. This underscores the value of integrating imaging with dynamic testing to improve diagnostic accuracy.

In Case 4, NDI was identified with an associated CLCN5 gene variant, raising the possibility of Dent disease. While classical descriptions of Dent disease focus on hypercalciuria and nephrocalcinosis, recent literature has expanded its phenotypic spectrum to include atypical presentations [10,11]. Our case contributes to this evolving understanding by demonstrating a predominantly polyuric presentation without overt classical features, thereby supporting the concept of genetic and phenotypic heterogeneity in NDI. Furthermore, the observed partial response to thiazide and amiloride therapy is consistent with established treatment paradigms described in prior studies [12,13].

In Case 5, primary polydipsia was diagnosed based on intact urine concentrating ability. Previous literature, particularly by Goldman et al., has linked primary polydipsia to behavioral factors and psychotropic medication use [14,15]. In our study, although the patient did not have a primary psychiatric disorder, lifestyle factors and intermittent medication use likely contributed, suggesting that primary polydipsia may be increasingly encountered outside traditional psychiatric settings. This aligns with emerging trends reported in recent studies.

Case 6 represents a particularly novel aspect of our series, where a pheochromocytoma presented with DI-like symptoms that resolved following tumor resection. While pheochromocytoma is classically associated with hypertension and catecholamine excess, its association with polyuria is rarely described. Existing literature suggests that catecholamine-mediated renal hemodynamic changes may impair urinary concentrating ability through increased renal blood flow, pressure natriuresis, and reduced responsiveness of the renal collecting ducts to vasopressin [16,17]. These mechanisms may result in transient polyuria and polydipsia, thereby mimicking DI. In our patient, DI was considered unlikely because there was no evidence of persistent vasopressin deficiency or renal resistance, and the symptoms resolved completely within a few days following adrenalectomy without desmopressin therapy. Our findings support this hypothesis and add to the limited body of evidence describing endocrine tumors as reversible mimics of DI, thereby expanding the differential diagnosis of polyuria-polydipsia syndrome.

Case 7 adds another important dimension to this spectrum by illustrating CDI in the context of postoperative and post-radiotherapy craniopharyngioma. Craniopharyngioma is well known to cause both anterior and posterior pituitary dysfunction due to tumor location, surgical intervention, and radiation-induced hypothalamic-pituitary damage. Our patient demonstrated evolving panhypopituitarism along with CDI, highlighting the long-term endocrine sequelae of hypothalamic-pituitary axis injury. This case reinforces the need for lifelong surveillance in such patients, as the delayed onset of CDI and multiple hormonal deficiencies is well documented [18]. Additionally, it underscores that polyuria in such settings should not be overlooked or attributed solely to other causes, as timely diagnosis and desmopressin therapy significantly improve quality of life.

In our series, the water deprivation test with desmopressin challenge remained central to diagnosis across all cases. This is consistent with traditional recommendations [1,4]. However, as highlighted in prior studies, this test is cumbersome and carries inherent risks. Recent literature has demonstrated that copeptin-based diagnostic algorithms offer superior accuracy, particularly in differentiating partial CDI from primary polydipsia [7,10,19]. Although copeptin-based testing was not routinely available because of resource constraints, our findings suggest that meticulous application of conventional diagnostic methods can facilitate appropriate etiological classification and guide targeted management in resource-limited settings. However, given the retrospective design, limited sample size, and absence of a comparator group, these observations should be interpreted as demonstrating clinical feasibility rather than establishing the diagnostic accuracy or superiority of classical testing.

Earlier studies, including those by Kalra et al., have reported mixed cohorts of CDI, NDI, and primary polydipsia, often with delayed diagnosis due to overlapping features [14]. Our series mirrors these findings but also extends them by demonstrating clear case-wise differentiation using a structured approach; highlighting partial CDI as a clinically significant intermediate entity; identifying a potential genetic NDI variant (CLCN5-related); reporting pheochromocytoma as a reversible DI mimic, which is rarely documented; and emphasizing primary polydipsia in a non-psychiatric, lifestyle-associated context. Thus, compared to existing literature, our study not only reinforces known diagnostic challenges but also contributes additional clinical insights and rare associations.

This study has several limitations. First, it was a retrospective single-center case series involving a small number of patients, which limits the generalizability of the findings. Second, advanced diagnostic modalities such as copeptin-based testing were not uniformly available, and diagnosis in most cases relied on conventional water deprivation and desmopressin challenge tests. Furthermore, complete dynamic diagnostic evaluation could not be uniformly performed in all patients owing to the retrospective nature of the study and real-world clinical constraints, which may have influenced diagnostic certainty in selected cases. Third, genetic testing could not be performed in all patients because of financial and logistical constraints, potentially limiting definitive etiological characterization in selected cases. In addition, the CLCN5 variant identified in Case 4 was classified as a VUS; in the absence of segregation analysis or functional validation, its pathogenic role remains speculative. Additionally, the descriptive nature of the study precludes assessment of diagnostic accuracy or comparative effectiveness of different diagnostic approaches. Nevertheless, the study reflects real-world clinical practice in a resource-limited setting and highlights the broad etiological spectrum of polyuria-polydipsia syndrome, emphasizing the continued utility of a structured diagnostic approach for accurate diagnosis and management.

Clinical implications

Our findings underscore that similar symptomatology does not equate to similar pathophysiology. Misclassification can lead to inappropriate therapy-particularly dangerous in cases such as primary polydipsia, where desmopressin may induce hyponatremia. Consistent with previous studies, our findings support the use of a multimodal diagnostic strategy integrating clinical, biochemical, dynamic, and radiological evaluation to facilitate appropriate etiological diagnosis and individualized management, particularly in resource-limited settings.

## Conclusions

In conclusion, this case series highlights the broad etiological spectrum of polyuria-polydipsia syndrome and demonstrates that, even in the absence of advanced biomarkers such as copeptin, accurate diagnosis can be achieved through systematic evaluation. Compared to existing literature, our study adds novel clinical scenarios and reinforces the importance of individualized, mechanism-based diagnosis in endocrine practice. Given the retrospective design and limited sample size, these findings should be interpreted as demonstrating the feasibility and clinical utility of this approach rather than establishing its diagnostic accuracy or generalizability. Larger prospective studies are warranted to validate these observations.
